# Synthesis of Superhydrophobic Cellulose Stearoyl Ester for Oil/Water Separation

**DOI:** 10.3390/nano12121964

**Published:** 2022-06-08

**Authors:** Qian Yang, Weiyin Su, Jianquan Hu, Yan Xu, Zhong Liu, Lanfeng Hui

**Affiliations:** Tianjin Key Laboratory of Pulp and Paper, Tianjin University of Science and Technology, Tianjin 300457, China; beyondmop@163.com (Q.Y.); 3592595957@163.com (W.S.); hujianquande@163.com (J.H.); xy199833@outlook.com (Y.X.)

**Keywords:** oil/water separation, adsorption, hydrophobic, membrane

## Abstract

Developing fluorine-free superhydrophobic and biodegradable materials for oil/water separation has already become an irresistible trend. In this paper, we designed two biopolymer oil/water separation routes based on cellulose stearoyl ester (CSE), which was obtained via the acylation reaction between dissolving pulp and stearoyl chloride homogeneously. The CSE showed a superhydrophobic property, which could selectively adsorb oil from the oil/water mixture. Additionally, the CSE was emulsified with an oxidized starch (OS) solution, and the resulting latex was used to impregnate commercial, filter base paper, finally obtaining a hydrophobic and oleophilic membrane. The SEM revealed the membrane had hierarchical micro/nanostructures, while the water contact angle indicated the low surface energy of the membrane, all of which were attributed to the CSE. The membrane had high strength and long durability due to the addition of OS/CSE, and the separation efficiency was more than 99% even after ten repeated uses.

## 1. Introduction

Oil is an important resource for human survival, but oil spill accidents often occur in the process of using oil, which not only causes energy waste but also causes harm to environmental safety [1,2]. With the improvement of environmental protection requirements, if the petrochemical and machinery industries are not able to quickly treat oily sewage, it will be a burden on production. Therefore, how to effectively treat oily wastewater is always a problem; therefore, it is a hot topic and difficult point in the research of scientists [3,4].

Among these advanced technologies, materials with superhydrophobic and superoleophilic surfaces can selectively absorb or filter oil from oil/water mixtures, which are usually called “oil-removing” types of materials [5,6,7]. Powdery substances, such as metallic oxide [8], mineral filler [9], and SiO_2_ nanoparticles [10], have unique advantages in thin oil spill absorption after hydrophobic modification. A number of researchers have recently investigated oil adsorption underwater with superhydrophobic particles. Stearic acid-modified CaCO_3_ can purify 99.6% of diesel [11], and the magnetic SiO_2_ nanocomposite particles modified by vinyltrimethoxysilane can adsorb 7.15 g/g of lubricating oil [10]. Furthermore, these as-prepared superhydrophobic powdered materials can be used to modify natural textiles [12], synthetic membranes [13], cellulose-based aerogels [14], or sponges [15,16] for oil/water mixture separation. Bagoole et al. [17] functionalized 3D graphene sponges with a high-elastic compression modulus that can adsorb as high as 3300 wt% of crude oil, which showed excellent adsorption–desorption properties. The superhydrophobic particle was flexible and highly efficient in the application of oil/water separation; however, most of them are expensive metal particles and should be modified using a silicone- or fluorine-based chemical, which may threaten human health and the environment [18]. Therefore, biodegradable, renewable, and cost-saving approaches are highly desired to fulfill efficient oil/water separation. Cellulose is the most suitable raw material, and acylation products of cellulose with long-chain alkyl hydrocarbons are superhydrophobic, biodegradable, and eco-friendly [19]. The degree of substitution [20], as well as the side-chain length [21] of the acylation reaction, can affect the hydrophobicity of cellulose-based ester. Zhang et al. [22] synthesized cellulose stearoyl ester and fabricated superhydrophobic paper via dip coating and spray coating. Li et al. [23] revealed the mechanism of a cellulose, ester-based superhydrophobic coating. The abovementioned studies achieved cellulose-based superhydrophobic surfaces, but they were not concerned with practical applications. To the best of our knowledge, using biomass-based CSE for oil/water separation via adsorption and filtration methods has not been reported.

In this article, versatile superhydrophobic cellulose stearoyl ester (CSE) was used for oil–water separation in two rationally modulated ways. CSE can not only selectively absorb oil underwater quickly but also can be used to assemble a hydrophobic and rough membrane to filter the oil from an oil/water mixture by adhering it to the fiber surface of filter base paper (FBP) under the emulsification of starch solution. Additionally, starch was commonly used in papermaking as a strengthening agent to enhance the strength of the substrate [24]. This study is the first to delve into the practical applications of CSE and test its properties, which might be a new generation in oil–water separation materials.

## 2. Materials and Methods

### 2.1. Materials

Bleached hardwood dissolving pulp was obtained from Sun Paper Industry Co., Ltd. (Jining, China). Oxidized starch was obtained from Huatai Group Co., Ltd. (Dongying, China). Filter base paper (base weight 155 g/m^2^) was obtained from Wanhao Group, Co., Ltd. (Weifang, China). Stearoyl chloride was purchased from Aladdin Co., Ltd. (Shanghai, China). *N,N*-dimethylacetamide (DMAC) was purchased from 3A chemicals Co., Ltd. (Shanghai, China), and sealed with molecular sieve desiccant. The moisture content was below 50 ppm. Anhydrous lithium chloride, triethylamine, and dichloromethane were bought from Fuchen Chemical Reagent Co., Ltd. (Tianjin, China). Methanol was purchased from Meryer Chemical Technology Co., Ltd. (Shanghai, China).

### 2.2. Preparation of CSE and OS/CSE Latex

Briefly, 1 g of vacuum-dried dissolving pulp was dissolved in 50 mL of DMAC, and 3.5 g of vacuum-dried LiCl solution [25] and 2.58 mL of triethylamine were added to the solution under magnetic stirring, and then 6.16 mL of stearoyl chloride was dropped into the solution. The reaction lasted for 6 h at 90 °C. Then, the solution was centrifuged at 8000 rpm for 10 min, and the sediment was completely washed with methanol and water. The resulting product, CSE, was vacuum-dried at 30 °C for 24 h [26]. The degree of substitution was measured as 1.18 via alkali hydrolysis-acid back titration [27]. Then, 1 g of CSE was added into a 3% gelatinized starch solution and stirred at 11,000 rpm by T 18, (IKA, Staufen im Breisgau, Germany). The OS/CSE latex was prepared for the impregnation of FBP, and the base weight of the fabricated OS/CSE@FBP membrane was 175 g/m^2^ after drying.

### 2.3. Characterization

Attenuated total reflectance Fourier transform infrared spectra (Bruker VERTEX 70, Rheinstetten, Germany) were used to record the chemical structure of CSE and OS/CSE@FBP at room temperature. The particle size of latex was measured by laser particle size analyzer (90 plus, Brookhaven, NY, USA). Surface morphologies were investigated using scanning electron microscopy (JSM-IT300LV, Hitachi, Tokyo, Japan) at an acceleration voltage of 10 kV. Energy-dispersive spectroscopy was measured by X-MaxN (Oxford Instruments, Oxford, Britain). The water contact angle was measured with 3 µL of deionized water by a dynamic contact angle measuring instrument (PGX, Fibro, Stockholm, Sweden). Pore size distribution of base paper and OS/CSE@FBP were measured by a capillary pore diameter measuring instrument (Porolux 100, Porometer NV, Nazareth, Belgium), the surface tension of infiltrating fluid was 16 mN/m.

Oil absorption behavior underwater was conducted as follows [28]: In the flask, pre-weighted dichloromethane (dyed with Sudan red) was poured into 100 mL of water, and then CSE was continually added into the flask under magnetic stirring until there was no free red droplet observed in the saturated, absorbed solution. The oil adsorption capacity was calculated based on the equation,$\;\mathrm{Adsorption}\;\mathrm{capacity}=M_{1}/M_{0}$, where M_0_ and M_1_ are the weight of CSE and pre-weighted dichloromethane, respectively. The experiment cycle was as follows: the compound ‘dichloromethane powder’ was washed with methanol under stirring conditions and then dried to repeat the adsorption [29].

The dichloromethane–water mixture was separated by an OS/CSE@FBP membrane, which was fixed between two glass tubes. Briefly, 20 mL of dichloromethane and 20 mL of water were mixed in a beaker and stirred for 5 min, and then added into the upper tube. After complete separation, the dichloromethane was collected from the receiving tube at the bottom. The oil/water separation was calculated by the equation, Eff=V/20×100%, where V is the volume of dichloromethane from the receiving tube at the bottom.

## 3. Results and Discussion

### 3.1. Preparation of CSE and OS/CSE@FBP Membrane

**Figure 1:**
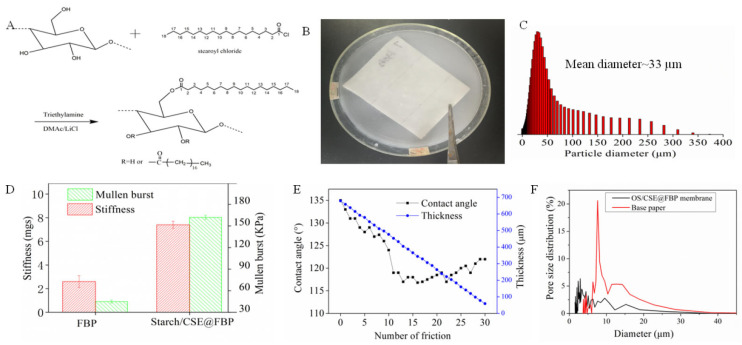
(**A**) synthesis route of CSE, (**B**) impregnation process of OS/CSE@FBP membrane, (**C**) particle size distribution of CSE, (**D**) stiffness and bursting strength of base paper, OS/CSE@FBP and (**E**) thickness and contact angle changes during friction, (**F**) pore size distribution of FBP and OS/CSE@FBP membrane.

The reaction of dissolving pulp and stearoyl chloride was performed in homogeneous conditions, and the molar ratio of glucose unit to acyl chloride was designed as 1:3. After the acylation reaction, parts of the cellulose hydroxyl group were converted into a new ester bond, which formed CSE with a long alkyl hydrocarbon structure. The synthesis route is shown in Figure 1A. CSE was emulsified in a gelatinized OS solution to form uniform latex, and the mean particle diameter of latex was 33 μm. The filter base paper was dipped into latex and dried in an oven and a smooth surface of the pristine base paper was covered by fine OS/CSE particles. The impregnation process and latex particle size distribution are shown in Figure 1B,C. The stiffness and Mullen burst of the membrane were important, because normally, a membrane should be embossed and pleated into a wavy shape and installed into a metal or plastic house to make a filter element. Starch was helpful in improving stiffness and Mullen burst. After being impregnated with latex, compared with FBP, the stiffness and Mullen burst of the OS/CSE@FBP membrane improved by 190% and 260%, respectively. The results are shown in Figure 1D. The friction resistance test was conducted by rubbing with commercial, 240-mesh sandpaper, back and forth 10 cm, 30 times under 50 g of weight. The paper scraps were ground out during the test and after 30 repetitions of the friction test (Figure 1E). The contact angle was always kept above 110°, even though the thickness decreased from 680 to 65 µm. This was attributed to the latex being deeply saturated in FBP and attached to a fiber surface by a strong, hydrogen-binding interaction of OS and cellulose. After impregnation of the OS/CSE latex, maximum pore size, mean pore size, and minimum pore size were all decreased (Table 1) because, after impregnation of OS/CSE latex, parts of pores of filtration base paper were blocked. The pore size distribution is shown in Figure 1F.

### 3.2. FTIR and EDS Analysis of CSE and OS/CSE@FBP Membrane

**Figure 2:**
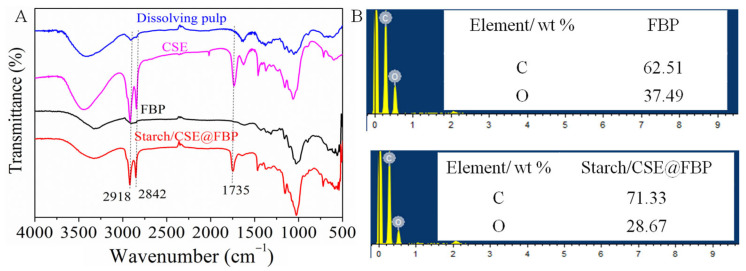
(**A**) FTIR spectra of dissolving pulp, CSE, FBP, and OS/CSE@FBP; (**B**) EDS spectra of FBP and OS/CSE@FBP.

The FTIR and EDS were explored to confirm the chemical structure changes. As can be seen from Figure 2A, compared with the spectra of dissolving pulp and CSE, the new sharp adsorption peak at 1735 cm^−1^ appeared, which belonged to the C=O stretching vibration of the ester group of CSE. The increased widths of the adsorption peaks at 2918, 2850, and 1465 cm^−1^ were associated with CH groups of cellulosic backbones and alkyl fatty hydrocarbon chains, while the asymmetric stretching vibration absorption peaks between 1060 and 1156 cm^−1^ were a C-O-C group of cellulose glucose unit [30]. After FBP was impregnated with OS/CSE latex, the new sharp adsorption peak at 1735 cm^−1^ appeared, which belonged to the C=O stretching vibration of the CSE–ester bond. As seen from EDS spectra in Figure 2B, the carbon content increased, and oxygen content decreased accordingly, compared with membrane and FBP, because the carbon content in grafted alkyl fatty hydrocarbon chains of CSE was higher than in the base paper. From both ATR-IR and EDS, we can conclude the successful impregnation of latex into filter base paper.

### 3.3. Wettability of CSE and OS/CSE@FBP Membrane

**Figure 3:**
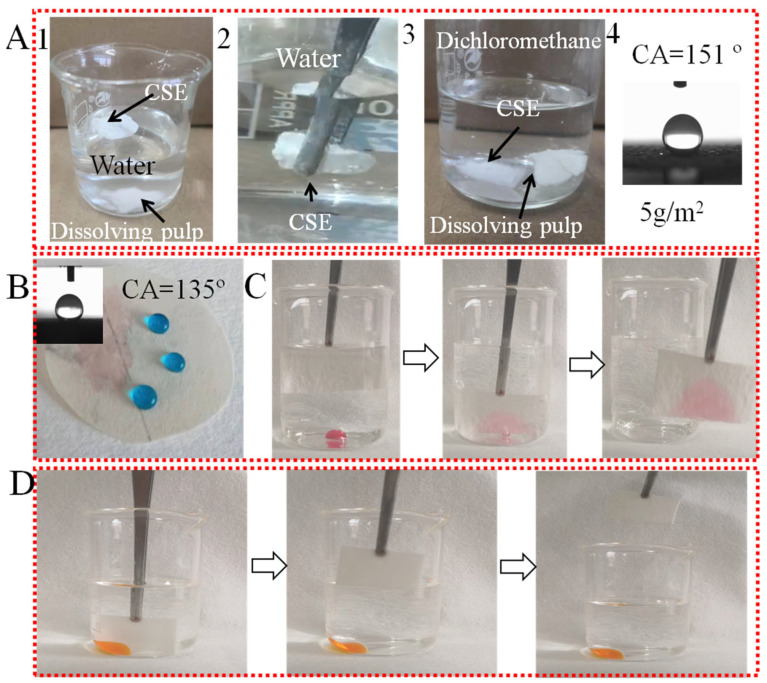
(**A**) Dissolving pulp sunk in water and dichloromethane, CSE floated on water and sank in dichloromethane, and the minimum deposition amount of 5 g/m^2^ CSE for superhydrophobicity; (**B**) WCA of OS/CSE@FBP membrane; (**C**) oleophilic in water; (**D**) hydrophobic in oil.

In Figure 3, the wettability of dissolving pulp, CSE, and OS/CSE@FBP membranes are investigated. As seen in Figure 3(A1), CSE had good buoyancy and floated on the water surface, while the native dissolving pulp sank into the water quickly. As shown in Figure 3(A2), when CSE was immersed in water by an external force, a silver mirror-reflection surface was observed, which was an interface phenomenon caused by a trapped air layer between the CSE and water [31]. The CSE floated up quickly once the external force was loosened, and it was found that no moisture was absorbed by the CSE. As shown in Figure 3(A3), both CSE and the dissolving pulp were easily wetted by dichloromethane. When CSE was in descent, the trapped air inside of the CSE was released into the solvent and formed bubbles. As shown in Figure 3(A4), CSE was ground and dispersed in ethanol, and deposited onto a hydrophilic filter membrane. After drying and curing, the CSE became agglomerated via hydrogen bonding. The minimum deposition amount to achieve a superhydrophobic effect was around 5 g/m^2^, which was lower than reported [32]. Based on these phenomena mentioned above, the CSE exhibited superhydrophobic and oleophilic properties after an acylation reaction.

The oil and water wettability behaviors of the OS/CSE@FBP membrane are shown in Figure 3B. The membrane was hydrophobic and oleophilic in the air, and the water droplet stood on the membrane, while the oil spread quickly. The water contact angle measured 135°, which was lower than the contact angle of the CSE, due to the weakening of the hydrophilic OS. As shown in Figure 3C, the membrane adsorbed a small amount of heavy oil dichloromethane underwater easily, thanks to its porous structure and hydrophobic properties. The water was repelled by the membrane but selectively adsorbed dichloromethane in the water. During the adsorption process, we saw bubbles released from within the membrane, which was the exchange of trapped air and dichloromethane. The hydrophobic membrane showed oleophilic properties underwater, however, as shown in Figure 3D. The membrane could not adsorb water (dyed in orange) in kerosene because when the membrane initially adsorbed kerosene, the oil layer formed a barrier of hydrophobicity, preventing water droplet adsorption.

### 3.4. Surface Morphologies of CSE and OS/CSE@FBP Membranes

**Figure 4:**
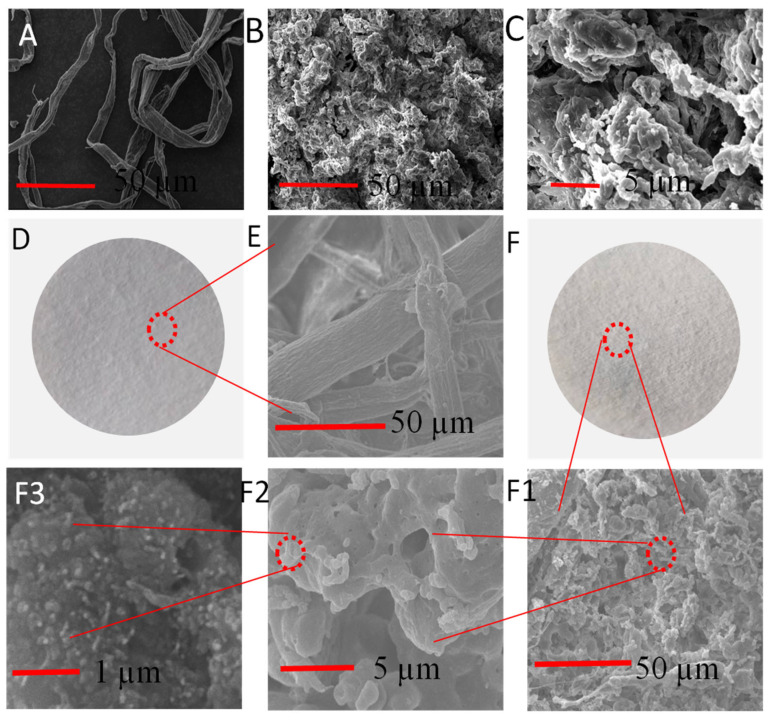
(**A**) SEM images of dissolving pulp; (**B**) CSE; (**C**) highly magnified CSE; (**D**) FBP; (**E**) highly magnified FBP; (**F**) OS/CSE@FBP membrane; (**F1**–**F3**) highly magnified membrane.

In order to investigate the surface morphology of dissolved pulp, CSE, and OS/CSE@FBP, a scanning electron microscopy (SEM) analysis was carried out. Figure 4A shows the original dissolving pulp was neat and fibrous, with a diameter of around 20 µm. However, ground CSE was a powdery, solid structure with a porous and rough surface after self-aggregation [33], as shown in Figure 4B. The structure was completely different from the initial fiber morphology, which means the long cellulose chain was totally destroyed in the acylation process. The obvious three-dimensional hierarchical structure of crater-like morphology was observed, and the presence of small elliptic particles could be seen clearly (Figure 4C).

FBP was typically smooth-surfaced, as shown in Figure 4D. It was loose and porous, and the fibers were interlaced and arranged into a network structure, which provided filter channels, as shown in Figure 4E. The surface of the OS/CSE@FBP membrane was a multidimensional structure and roughened by saturation of OS/CSE latex. The accumulated CSE particles were caused by interception of the porous network structure of FBP and the adhesion of starch, as shown in Figure 4(F1,F2). At the outer layer of large particles, a large number of nanosized particles could be seen (Figure 4F3), which further magnifies the surface roughness and hydrophobicity of the membrane.

### 3.5. Oil Adsorption Analysis of CSE

**Figure 5:**
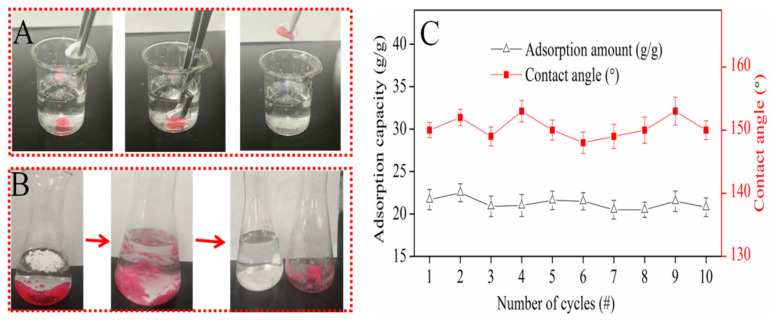
(**A**) CSE picks up oil underwater; (**B**) adsorption dichloromethane process; (**C**) recycling adsorption performance.

In order to investigate the potential application of oil/water mixture separation via adsorption, an external force was applied to bring the superhydrophobic CSE in contact with dichloromethane underwater. The operation process can be seen in Figure 5A. Bubbles came out of the CSE, and the CSE turned red directly in a second. When the solid was taken out, there was no liquid dropped, which meant the resulting solid was not only superhydrophobic–oleophilic but also able to clean up oil from water due to the porous structure.

As seen in Figure 5B, with the aim to evaluate the maximum oil adsorption capacity of CSE further, a pre-measured weight of dichloromethane was injected into the water. The ground CSE powder was continuously added until there was no free dichloromethane seen in the water. The CSE became agglomerated when it adsorbed dichloromethane. After filtration, the water seemed transparent, and no red oil was observed. The residuals were washed with methanol and dried in the oven to reuse for oil adsorption. This result is shown in Figure 5C. During the recycling process, the CSE adsorption capacity stayed almost constant at 21~23 g/g and resulted in transparent water. There was a slight loss of water each time, probably trapped by the ‘oil–CSE compound substance’, which indicated the CSE can fully and selectively adsorb oil underwater by capillary action. After it was dried in the oven, the CSE powder was deposited onto a hydrophilic membrane in a vacuum, and the water contact angle was tested. The result showed that during the recycling process, the CSE still showed hydrophobic properties, and the water contact angle data were always between 145 and 155°.

### 3.6. Oil–Water Separation of OS/CSE@FBP Membrane

**Figure 6:**
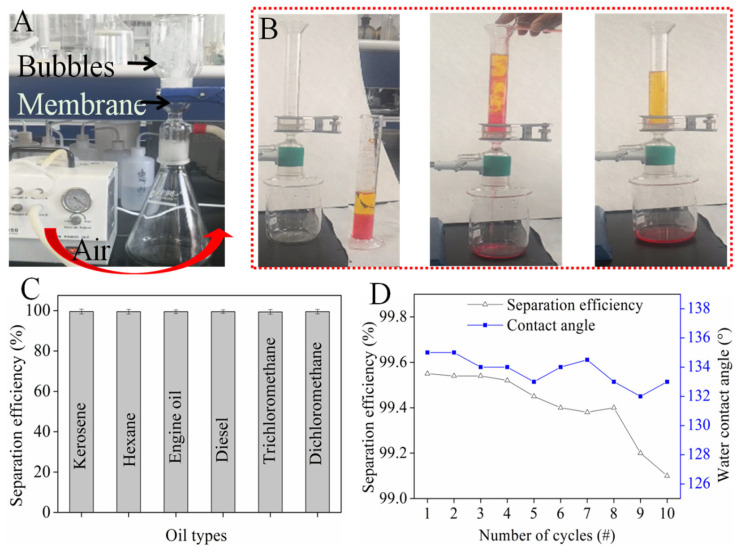
(**A**) Air permeability of membrane; (**B**) oil/water separation process; (**C**) separation efficiency of various oil–water mixture; (**D**) separation efficiency and contact angle of repeated tests.

The permeability of the oil/water separation membrane was a key parameter, which determined the oil flux [34]. As shown in Figure 6A, 250 mL of distilled water (10 cm height) was added to the suction flask. The membrane was fixed in the middle and the pressure on the membrane was 0.98 kPa. There was no water leakage for 48 h because the membrane was hydrophobic. It was found when air was pumped in from the bottom, a large number of bubbles were generated in the upper bottle, which indicated that the membrane was highly permeable after being impregnated with OS/CSE latex.

The separation process is shown in Figure 6B. Water (dyed in methyl orange) was mixed with dichloromethane (dyed with Sudan red) and poured down from above the device. The water phase with low gravity contacted the membrane first, and then the dichloromethane passed through the water phase and touched the membrane surface, instantly penetrating through the membrane in 9 s. The water phase was repelled and stayed on top of the membrane. The OS/CSE@FBP membrane separated various types of oil/water mixture, and the separation efficiency rates, shown in Figure 6C, were all higher than 99%.

The durability of OS/CSE@FBP was evaluated by measuring the contact angle and oil-separation efficiency after each separation. As shown in Figure 6D, after 10 cycles of testing, the membrane still maintained more than 99% of the separation efficiency, and the contact angle always remained above 130°, indicating that the stability and durability of the membrane were well-retained.

## 4. Conclusions

In summary, superhydrophobic and oleophilic CSE was synthesized via acylation reaction of cellulose and stearyl chloride homogeneously, which could separate an oil/water mixture in the forms of adsorption and filtration. CSE exhibited a high oil adsorption capacity underwater as well as high reusability performance. CSE was emulsified with an oxidized starch solution and impregnated with commercial filter base paper, and a hydrophobic and oleophilic OS/CSE@FBP membrane was obtained, which can also adsorb oil underwater. Importantly, the as-prepared membrane can separate various types of oil/water mixtures, and the separation efficiency rates were all higher than 99% and exhibited good reusability performance. The renewable and biodegradable CSE is a potentially valuable material for oil–water separation.

## Figures and Tables

**Table 1:** Pore sizes of base paper and OS/CSE@FBP membrane.

	Unit	Filter Base Paper	OS/CSE@FBP Membrane
Maximum pore size	µm	38.1	25.3
Mean pore size	µm	13.7	10.4
Minimum pore size	µm	4.6	1.6

## Data Availability

The data are available on reasonable request from the corresponding author.
